# A CNN-BiLSTM-GRU and attention-integrated hybrid network for epileptic seizure detection

**DOI:** 10.3389/fnins.2026.1909680

**Published:** 2026-08-10

**Authors:** Xingran Wang, Tinghao Gong, Xuejia Li, Tianhua Lin

**Affiliations:** School of Management Science and Information Engineering, Hebei University of Economics and Business, Shijiazhuang, China

**Keywords:** attention mechanism, bidirectional long short-term memory, deep learning, EEG signals, epileptic seizure detection, gated recurrent unit, one-dimensional convolutional neural network

## Abstract

**Introduction:**

Epilepsy is a common neurological disease, and accurate seizure detection is essential for clinical monitoring and scientific treatment. This study aims to construct an effective intelligent detection model to achieve precise automatic identification of epileptic EEG signals and assist clinical medical decisions.

**Methods:**

To capture subtle local waveform variations and suppress redundant noise interference in EEG signals, this study adopts one-dimensional convolutional neural network (1D-CNN) layers for adaptive local feature extraction and a lightweight global temporal soft attention mechanism for critical feature enhancement. A hybrid classification model based on bidirectional long short-term memory (Bi-LSTM) and gated recurrent unit (GRU) is proposed for the binary classification of epileptic EEG signals. The synthetic minority oversampling technique (SMOTE) is applied only to the training data within each cross-validation fold to alleviate the class imbalance problem of EEG datasets.

**Results:**

The proposed hybrid model achieves a binary classification accuracy of 99.23%, while delivering an especially balanced sensitivity (99.29%) and specificity (99.34%), with a difference (∆ Sens–Spec) of only 0.05%, verified on the public UCI epileptic seizure recognition data set.

**Discussion:**

The CNN-Bi-LSTM-GRU and attention-integrated hybrid network can effectively distinguish seizure and non-seizure EEG signals. And a nearly equal sensitivity and specificity suggests robust and unbiased classification. Which is critical for clinical deployment. The proposed method achieves competitive performance compared with most recent mainstream algorithms, which can offer a potential automated detection reference to assist clinical analysis of epilepsy EEG signals.

## 1 Introduction

Epilepsy is one type of neurological diseases characterized by abnormal electrical discharges from brain neurons, that is usually accompanied by recurrent seizures without a known cause (Ghosh and Sinha, 2026; Guranda et al., 2025). Its characteristics of suddenness and unpredictability not only threaten the life safety of patients, but also bring psychological and social burden. EEG signals directly reflect the electrical activity of the cerebral cortex and contain rich electrophysiological characteristics (Gkintoni et al., 2025; Walla, 2025). It is an important mean of non-invasive monitoring of epileptic seizures, playing a key role in the clinical diagnosis of epilepsy. Since EEG signals exhibit characteristics such as nonlinearity, intense noise, high dimensionality, and strong time series, analyzing the entire EEG recordings manually is not only extremely time-consuming but also presents problems such as intense subjectivity and poor repeatability. Automatic detection of epileptic seizures based on EEG signals has become an important method for auxiliary diagnosis in the clinical field. Therefore, the efficient and accurate detection of epileptic seizures based on EEG and computational methods brings challenges to traditional machine learning models and has become a core task in this research area.

With the development of deep learning techniques (Diab et al., 2025), the research about the feature extraction and classification of EEG signals has been explored actively. Several results have demonstrated that deep learning techniques were highly effective for EEG data analysis, such as fatigue state detection (Dello Iacono et al., 2025; Yang et al., 2025), person identification (Cao et al., 2014) and the detection of various nervous disorders (Amital et al., 2025; Nassehi et al., 2026). In summary, these studies demonstrate that these deep learning methods based on EEG data are effective in identifying complex patterns of neural data. For example, the convolutional neural network (CNN) has been demonstrated to be highly effective at extracting spatial features as one of the most common deep learning models, showing outstanding performance in many applications (Radenovic et al., 2017). However, CNNs have limitations in retaining memory of previous temporal patterns, making it a challenge to directly learn key features of EEG signals. The recurrent neural network (RNN)(Choi et al., 2017) can effectively capture temporal features from data to make up for the shortage of CNNs in learning long-term dependency relationships. LSTM (Hanson et al., 2017) and GRU (Niu et al., 2023) are both improved versions of RNNs that can not only solve the gradient vanishing and explosion problem, which often arise in traditional RNNs during long-sequence modeling, but also model sequences in terms of time.

Based on characteristics of these above deep learning methods, in recent years, researchers have conducted many studies on their application in epileptic seizure detection, which have promoted clinical application (Qamar et al., 2025) (Tan et al., 2025). For example, many researchers have attempted to extract both temporal features and spatial features by developing novel CNN–LSTM hybrid models, and some studies have demonstrated the feasibility of this approach (Sun et al., 2019). Recently, three types of model (CNN, LSTM, and a CNN–LSTM hybrid model) have been tried for epileptic seizure detection (Hermawan et al., 2024). These results have showed that the CNN excelled in spatial feature extraction, the LSTM performed best in the temporal dynamic capturing, and the hybrid model combined both of these advantages. These findings confirm that LSTM can address CNN’s limitations in modeling long-term temporal dependency relationships, resulting in a better detection performance. Considering the bidirectionality of specific sequences, a hybrid model integrated with Bi-LSTM network was proposed by Dong and Wen (2024), which demonstrating the potential of Bi-LSTM for real-time epileptic seizure monitoring.

As one specialized RNNs, the GRU network provides benefits in both structure and computational efficiency compared to other RNNs like LSTMs (Ayzel and Heistermann, 2021; Yu et al., 2021). An automated system for epileptic seizure detection based on a bidirectional GRU network was proposed by Zhang et al. (2022), which exhibited good performance. And a CNN–GRU hybrid model was proposed by Abgeena and Garg (2023), which was based on the ability of GRU to explore temporal correlations, for emotion recognition from EEG signals. The results demonstrated that this hybrid model is outstanding among most of the current competitive hybrid deep learning models in terms of performance.

These studies have fully demonstrated the enormous potential of various deep learning models in the detection of epileptic seizures based on EEG signals, however, still remain certain limitations. For instance, a 1D CNN-LSTM model was proposed (Xu et al., 2020), which was verified on the public UCI epileptic seizure recognition data set, achieving high recognition accuracies of 99.39% on the binary class epileptic seizure, without sensitivity and specificity analysis. Comparing these results with other deep learning methods has showed the superiority of this proposed method. However, CNNs perform well at capturing spatial features, but it is difficult to fully model the dynamic dependency relationships of long-term series, and for CNN-RNN hybrid models, balancing the ability to capture long-range temporal dynamic dependency relationships with computational efficiency still remains a challenge. Considering the issues mentioned above, this paper proposed a hybrid network model based on CNN-BiLSTM-GRU and attention mechanism for epileptic seizure detection in EEG signals.

## 2 Methods

### 2.1 EEG dataset

**Figure 1:**
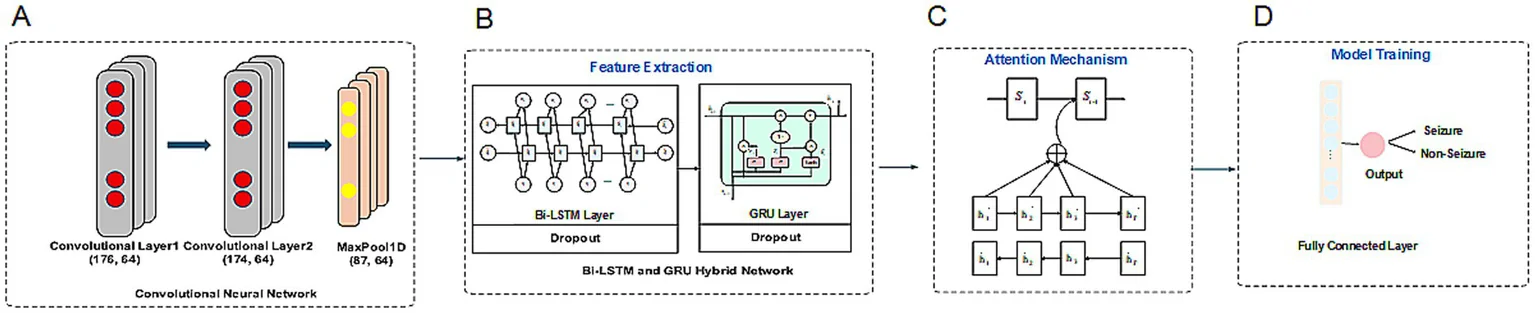
Framework of the hybrid network model based on CNN-Bi-LSTM-GRU and attention mechanism for epileptic seizure and no seizure detection in EEG signals. **(A)** 1D-CNN: The module for extracting local spatial waveform features of raw EEG sequences; **(B)** Bi-LSTM and GRU: The model for feature extraction and classification; **(C)** Attention mechanism: The module for adaptively assigning weight coefficients to different temporal features to highlight critical epileptic characteristic information. **(D)** Fully Connected Layer: the module for feature aggregation and generating final classification results to distinguish seizure and non-seizure EEG samples.

This study chooses one dataset for epileptic seizure detection, that is obtained from the UCI Machine Learning Library and is from Bonn University Epilepsy Seizure Dataset after preprocessing and reorganizing (Andrzejak et al., 2001). The dataset contains 500 EEG segments, divided equally into 5 subsets. These segments are extracted from longer multi-channel EEG recordings following the removal of muscle and eye movement artifacts. Each EEG segment is sampled at 173.61 Hz over a 23.6-s period, resulting in 4097 data points. Each point corresponds to the EEG signal amplitude at a specific time. Each EEG segment is divided into 23 slices (lasting about 1 s), including 178 data points. These 5 sub-datasets are labeled into 5 distinct categories, each containing 2,300 signal slices. These 5 subsets are originally labeled from 1 to 5, representing epileptic seizure activities, EEG from the tumor-localized area, EEG from the healthy brain area (not tumor located area), EEG recorded during the eyes-closed state, and EEG recorded during the eyes-open state, respectively. The complete dataset is accessible via the following link: https://www.kaggle.com/datasets/harunshimanto/epileptic-seizure-recognition (See Figure 1)

### 2.2 Data preprocessing

**Figure 2:**
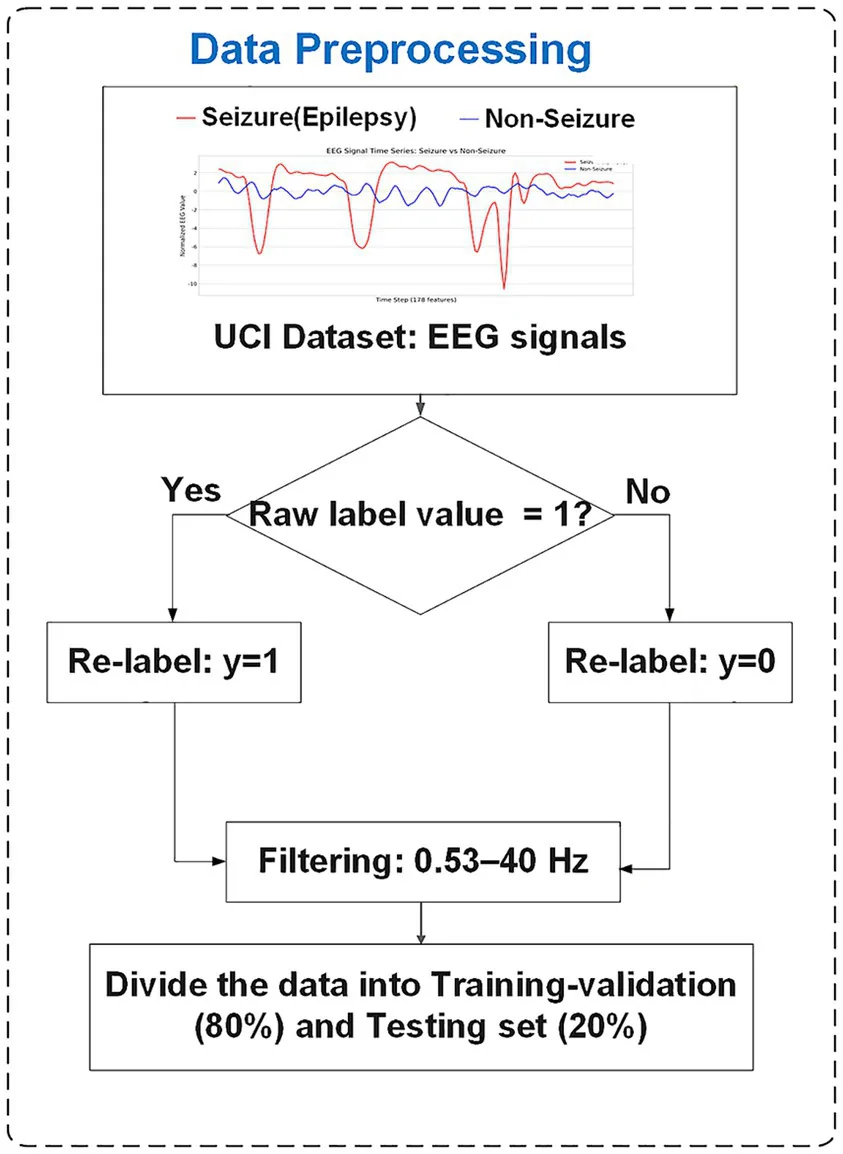
Data preprocessing includes relabeled, filtering, and data distribution.

In this study, these specific steps of data preprocessing are illustrated in Figure 2, to ensure the quality and consistency. The seizure detection task is defined as a binary classification problem, so that the UCI dataset is relabeled into a binary format: samples with the original label 1 are identified as seizure data and relabeled as y = 1, while samples with the original labels 2, 3, 4, and 5 are considered non-seizure data and relabeled as y = 0. As the key EEG information related to epileptic activity is mainly below 40 Hz frequency, a 4th-order Butterworth band-pass filter with a passband of 0.53–40 Hz is applied to remove irrelevant frequency components. Finally, the data was divided into training–validation set (80%) and testing set (20%).

### 2.3 Model design

As shown in Figure 1, this study applies a hybrid network model based on a 1D-CNN, Bi-LSTM and a GRU for epileptic seizure detection in EEG signals. These preprocessed EEG slices serve as input, each containing 178 sequential feature points. Firstly, as shown in Figure 1A, two one-dimensional convolutional layers (1D-CNN) are deployed to adaptively extract local waveform features and subtle mutation characteristics from raw EEG sequences, which avoids manual feature engineering and obtains high-quality shallow feature representations. The extracted features are then fed into the subsequent temporal modeling module. As shown in Figure 1B, the Bi-LSTM layer is applied to fully capture the forward and backward temporal dependency relationships of EEG signals, followed by the GRU layer to extract key temporal features. Both the Bi-LSTM and GRU layers utilize the tanh activation function and are each accompanied by a dropout layer with the parameter of 0.5 to prevent overfitting. Subsequently, as shown in Figure 1C, an attention mechanism is introduced to adaptively assign weight coefficients to different temporal features, enhancing critical epileptic characteristic information and suppressing redundant noise. Finally, as shown in Figure 1D, two fully connected layers are appended to the end of the model: the first layer contains 64 neurons for feature integration with the ReLU activation function, and the second layer serves as the output layer with the sigmoid activation function, containing a single neuron. The final output represents the probability of a seizure, with a decision threshold set at 0.5. Compared to a conventional neural network framework, this hybrid model integrated with CNN, Bi-LSTM and GRU modules can not only effectively model the millisecond temporal dynamic characteristics of EEG signals but also accurately capture the characteristic rhythm evolution process of epileptic seizures. It is achieved through the collaborative work of CNN, Bi-LSTM and GRU, providing a more reliable analytical basis for the epileptic seizure detection in EEG signals. These model-related parameters are summarized in Table 1.

**Table 1:** Model-related parameters.

Layer	Kernel size/hidden units	Output channels	Activation function
Conv1D	Kernel length = 3	64	ReLU
Conv1D	Kernel length = 3	64	ReLU
MaxPooling1D	Pool size = 2	64	—
Dropout	Rate = 0.3	—	—
Bi-LSTM	128 hidden units return_sequences = True	—	tanh
Dropout	Drop rate = 0.5	—	—
GRU	128 hidden units return_sequences = True	—	tanh
Attention Layer	Trainable weight attention aggregation	—	tanh+ softmax
Fully connected layer	64 neurons	—	ReLU
Output layer	1 neuron	—	sigmoid

#### 2.3.1 One-dimensional convolutional neural network (1D-CNN) layers

Given the one-dimensional temporal characteristics of electroencephalogram (EEG) signals, this model adopts two one-dimensional convolutional layers (Conv1D) as the core local feature extraction module for subsequent temporal feature learning. As typical time-series data, epileptic EEG signals usually exhibit subtle local waveform mutations and rhythmic abnormalities during seizure onset, which can be effectively captured by 1D-CNN through sliding convolution operations. The core calculation formula of the 1D convolution operation is defined as Equation (1):

$$y(i)=\sum_{k=0}^{K-1}w_{k}\cdot x(i+k)+b$$ (1)

where $X_{\left(i\right)}$ represents the original EEG sequence, $w_{k}$ denotes the convolution kernel weight, K is the kernel size, b is the bias term, and $Y_{\left(i\right)}$ refers to the output feature value after convolution.

The specific network configurations are as follows. The first Conv1D layer employs 64 convolution kernels of size 3 with ReLU activation to traverse the 178-dimensional EEG signals. It maps raw time-series signals into a high-dimensional feature space and extracts shallow local features, such as waveform fluctuations and local peak changes. The second Conv1D layer maintains consistent parameters, including 64 kernels of size 3 and ReLU activation, to further mine deep discriminative features and strengthen the representation of seizure-related local patterns.

The primary advantage of the 1D-CNN module is its capability of adaptive local feature learning without manual feature engineering. It effectively avoids the inefficiency and subjectivity of traditional handcrafted feature methods and provides high-quality spatial-local feature representations for the subsequent BiLSTM and GRU temporal modeling layers.

#### 2.3.2 Bi-LSTM layer

LSTM is one special type of RNN, primarily created to solve the gradient vanishing and explosion problems in long-sequence training (Chen et al., 2024b; Iqbal et al., 2025). Compared with conventional RNNs, LSTM demonstrates better performance on longer sequences. An LSTM unit is equipped with three gates (forget, input, output) that regulate information flow. These gates can learn which parts of the sequence should be retained as important information and which should be eliminated, ultimately achieving the transmission of relevant information along long sequences for prediction tasks.

As an improved RNN, LSTM can capture information over longer-distance sequences. However, both LSTM and conventional RNNs have a limitation: when processing sequences from left to right, the later information tends to be more important than the earlier. To solve this issue, the bidirectional LSTM is proposed, which can execute LSTM once from left to right, then execute LSTM again from right to left, finally, combine the two results.

Bi-LSTM is a structural extension of LSTM (Saha et al., 2026), first introduced by Schuster and Paliwal in 1997. A Bi-LSTM processes sequences bidirectionally using two independent hidden layers: a forward LSTM and a backward LSTM, with their combined outputs serving as the input to the output layer. The calculation is defined by the following Equations (2)–(4):


$$\vec{h_{t}}=f(\vec{W}x_{t}+\vec{U}h_{t-1})$$ (2)

$$\overset{\leftarrow }{h_{t}}=f(\overset{\leftarrow }{W}x_{t}+\overset{\leftarrow }{U}h_{t+1})$$ (3)

$$o_{t}=g(V\vec{h_{t}}+V^{\prime }\overset{\leftarrow }{h_{t}})$$ (4)

W , U , V and $V^{\prime }$ represent the weights; $X_{t}$ indicates the input of the current unit; $h_{\left(t-1\right)}$ and $h_{\left(t+1\right)}$ are the outputs of the previous and next units, respectively. The Bi-LSTM network is a deep RNN structure that possesses both forward and backward temporal processing capabilities. For the detection of epileptic seizures, Bi-LSTM can effectively capture the global temporal dependency relationships of EEG signals. Bi-LSTM serves as the key component for feature extraction in the proposed model, providing sequence embeddings with strong discriminative power for subsequent classifiers.

#### 2.3.3 GRU layer

The GRU is also an improved RNN structure, proposed by Cho et al.(K. Cho et al., 2014) in 2014, that was designed not only to solve the gradient vanishing and explosion problems but also to simplify the network structure, reduce computational complexity, and ensure the expressive power. Compared with conventional RNNs, the GRU dynamically controls the flow of information through the update gate and reset gate.

About the working mechanism of the GRU, the update gate is used to control the amount of information in the current hidden state which is inherited from previous time steps. The calculation is defined by the following Equation (5):


$$z_{t}=\sigma (W_{z}\times [h_{t-1},x_{t}])$$ (5)

$X_{t}$ is the input of current time step, $h_{t-1}$ is the hidden state of the previous time step, $W_{z}$ represents the weight matrix, and σ represents the sigmoid activation function. The $Z_{t}$ value of the update gate ranges between 0 and 1, which can control the degree of retention of past information.

The reset gate determines the proportion of past information to be discarded and it is computed using Equation (6):


$$r_{t}=\sigma (W_{r}\times [h_{t-1},x_{t}])$$ (6)

Through this gate, the model can flexibly decide whether to ignore certain unrelated information in the old state, based on the current input.

When obtaining the candidate hidden state $\tilde{h_{t}}$ of the current time step, the GRU applies the reset gate to the historical hidden state and participates in the generation of the candidate state together with the current input, as expressed in Equation (7):


$$\tilde{h_{t}}=\tanh(W\;[r_{t}\times h_{t-1},x_{t}])$$ (7)

Finally, the current hidden state $\tilde{h_{t}}$ is obtained by a weighted combination of the candidate hidden state $\tilde{h_{t}}$ and the previous hidden state $h_{t-1}$ , as shown in Equation (8):


$$h_{t}=(1-z_{t})\times h_{t-1}+z_{t}\times \tilde{h_{t}}$$ (8)

This structure allows the model to flexibly adjust the reliance on new and past information at different time steps.

With the simple and efficient structure, GRU can not only retain the temporal characteristics when processing EEG signals related to epilepsy but also prevent overfitting of the model and difficulties in training. In this study, the GRU layer, as a deep feature integration module after the Bi-LSTM layer, effectively compresses the presequential temporal features. While controlling the computational cost, it provides stable and discriminative results for the following detection of epileptic seizures in the fully connected layer (Chen et al., 2024a; Gao et al., 2022).

#### 2.3.4 Dropout layer

Dropout is a regularization strategy applied to prevent overfitting in neural networks (Li et al., 2023). During the training, it will shield some neurons according to the preset probability, preventing the model from overly relying on some units and thereby reducing the coupling degree between features. This method enhances the adaptability of the model to new data. In this experiment, the dropout rate is set as 0.5.

#### 2.3.5 Attention mechanism layer

To address the problem of mixed redundant noise and insufficient weighting of critical effective features in EEG signals, a global temporal soft attention mechanism is embedded after the GRU layer. This module can adaptively assign different weights to temporal features, strengthen subtle pre-seizure characteristic information, suppress irrelevant noise interference, and further improve the model’s sensitivity to faint epileptic precursors.

This module adopts a simplified Bahdanau additive attention mechanism, which matches the self-defined Attention Layer structure in experimental codes. The computational procedures are expressed by the following Equations (9)–(11):


$$e_{t}=v^{T}\tanh(Wh_{t}+b)$$ (9)

$$\alpha_{t}=\text{softmax}(e_{t})=\frac{\exp(e_{t})}{\sum_{i=1}^{T}\exp(e_{i})}$$ (10)

$$\tilde{h}=\sum_{t=1}^{T}\alpha_{t}h_{t}$$ (11)

where $h_{t}$ denotes the temporal feature vector output by the GRU layer at time step t; W and v represent trainable weight matrices; b is the bias; $e_{t}$ is the preliminary attention score; $\alpha_{t}$ refers to the normalized attention weight corresponding to each temporal feature; and $\tilde{h}$ is the final attention-enhanced feature after weighted aggregation.

The module first receives temporal feature matrices output from the GRU network. Each feature vector undergoes linear transformation and tanh activation to acquire preliminary attention scores. Subsequent softmax normalization constrains all weights to sum up to one, endowing seizure-related time segments with higher weights and suppressing irrelevant noise data. Ultimately, weighted fusion is conducted to obtain enhanced features, which are transmitted into fully-connected layers for final classification.

Compared with complex multi-head attention variants, this lightweight additive attention structure has lower computational complexity and fewer training parameters. It effectively avoids overfitting and difficult convergence problems, making it more suitable for automatic epileptic seizure detection based on low-sampling-rate EEG signals (Yao et al., 2024).

#### 2.3.6 Fully connected layer

The fully connected (FC) layer is a basic and widely used component in deep neural networks. It can combine and map the features output by the previous layer to capture the global relationships among different features. Since each neuron in the layer remains connected to all nodes in the previous layer, this is why it is called a ‘fully connected layer’. Given an input vector x, its output can be expressed in Equation (12):


$$y=f(W_{x}+b)$$ (12)

Here, y represents the output vector, W represents the weight matrix, b represents the bias term, and f() is a nonlinear activation function. This process not only performs the linear transformation of features, but also introduces nonlinear expression capabilities, which are used for further abstraction and reconfiguration of information.

#### 2.3.7 Tanh activation function

The tanh activation function is the default activation function used in both Bi-LSTM and GRU models. As a gated activation function, its output range is [−1, 1], which helps regulate the flow of gradients. The function is given as Equation (13):


$$\tanh(x)=\frac{e^{x}-e^{-x}}{e^{x}+e^{-x}}$$ (13)

#### 2.3.8 ReLU activation function

The ReLU function is an extensively employed activation function in CNNs. It can help introduce nonlinearity and sparsity to the network, while also preventing the gradient vanishing problem. The function is given as Equation (14):


f(x)=max(0,x) (14)

The result of max(0,x) is the maximum of 0 and the input value x .

#### 2.3.9 Sigmoid activation function

The sigmoid activation function uniformly compresses input values into the range of (0, 1), which is commonly applied as the activation function in the output layer for binary classification tasks. Its output represents the probability of a sample belonging to a specific category. The calculation is defined by the following Equation (15):


$$\sigma (x)=\frac{1}{1+\exp(-x)}$$ (15)

Here, x represents the input value, σ(x) represents the output probability value, and exp represents the base value of the natural logarithm.

### 2.4 Model evaluation

The performance of this model was evaluated by stratified 10-fold cross-validation according to these comprehensive classification metrics: average accuracy (Acc), sensitivity (Sen), specificity (Spe), F1-score, Matthews Correlation Coefficient (MCC), Area Under Receiver Operating Characteristic Curve (AUROC), and Area Under Precision-Recall Curve (PR-AUC). These indicators collectively quantify the epilepsy seizure detection capability of this proposed model, as well as its potential for clinical application.

Accuracy represents the percentage of correct classifications over all samples. The calculation is defined by the following Equation (16):


$$\mathrm{Acc}=\frac{\mathrm{TP}+\mathrm{TN}}{\mathrm{TP}+\mathrm{FN}+\mathrm{TN}+\mathrm{FP}}\times 100\%$$ (16)

Sensitivity represents the percentage of correctly predicted seizure samples to the total actual seizure samples. The calculation is defined by the following Equation (17):


$$\mathrm{Sen}=\frac{\mathrm{TP}}{\mathrm{TP}+\mathrm{FN}}\times 100\%$$ (17)

Specificity represents the percentage of correctly predicted non-seizure samples to the total actual non-seizure samples. The calculation is defined by the following Equation (18):


$$\mathrm{Spe}=\frac{\mathrm{TN}}{\mathrm{TN}+\mathrm{FP}}\times 100\%$$ (18)

F1-score is the harmonic mean of precision and recall (same as Sen ), balancing false positive and false negative errors to mitigate evaluation bias caused by class imbalance. Precision denotes the proportion of true positives among all samples predicted as seizure. The F1-score ranges from 0 to 1, where a value approaching 1 signifies superior balanced detection performance. The formulas for precision and F1-score are Equations (19) and (20) respectively:


$$\text{Precision}=\frac{\mathrm{TP}}{\mathrm{TP}+\mathrm{FP}}\times 100\%$$ (19)

$$F1-\text{score}=2\times \frac{\text{Precision}\times \text{Recall}}{\text{Precision}+\text{Recall}\;}\times 100\%$$ (20)

MCC integrates all four confusion matrix entries (TP, TN, FP, FN), delivering robust and unbiased assessment even on severely imbalanced medical datasets. Its range spans from −1 to 1: 1 denotes perfect classification, 0 corresponds to random guessing, and −1 indicates fully reversed predictions. The MCC formula is given as Equation (21):


$$\mathrm{MCC}=\frac{\mathrm{TP}\times \mathrm{TN}-\mathrm{FP}\times \mathrm{FN}}{\sqrt{\left(\mathrm{TP}+\mathrm{FP})(\mathrm{TP}+\mathrm{FN})(\mathrm{TN}+\mathrm{FP})(\mathrm{TN}+\mathrm{FN}\right)}}\times 100\%$$ (21)

AUROC evaluates the global discriminative capacity of probability outputs across all classification thresholds. The ROC curve plots the true positive rate (sensitivity) against the false positive rate (1 − *Spe*). An AUROC value close to 1 indicates excellent separation between seizure and non-seizure EEG signals, while a value of 0.5 equals random discrimination.

PR-AUC serves as a complementary metric to AUROC for imbalanced seizure detection tasks. The PR curve plots precision against recall, focusing exclusively on the predictive quality of the minority seizure class. PR-AUC is more sensitive to false positive errors than AUROC and avoids overly optimistic performance estimates when seizure samples account for a small fraction of the dataset, providing a more realistic reflection of clinical practicality.

The terms in these above Equations (16)–(22) are defined as follows: TP is the count of correctly identified seizure samples; FN is the count of seizure samples incorrectly identified as non-seizure; TN is the count of correctly identified non-seizure samples; and FP is the count of non-seizure samples incorrectly identified as seizure.

### 2.5 Model training

**Figure 3:**
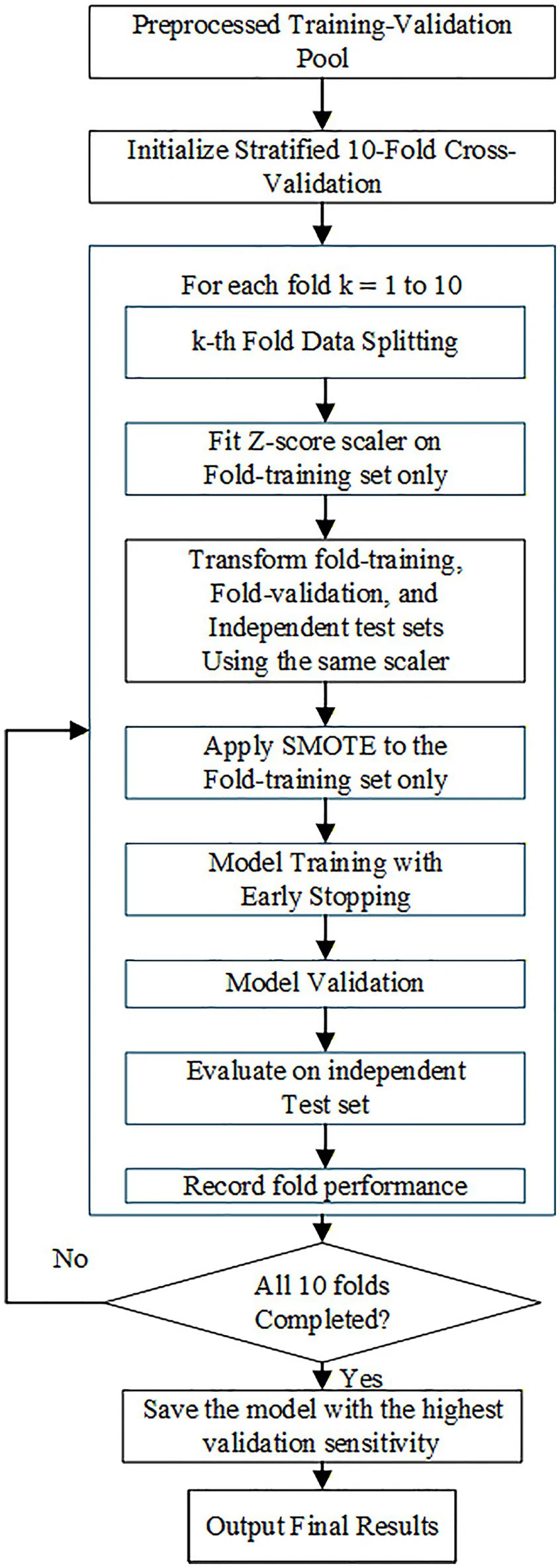
The 10-fold cross-validation flow diagram for epileptic seizure detection.

In this study, a 10-fold cross-validation approach was applied to avoid fluctuations in results caused by random partitioning, and the workflow is showed in Figure 3. In each fold cross-validation, the training set and validation set were firstly splitted from the preprocessed training - validation set, and the test set remained independent.

Then, to reduce the adverse impact of feature value range differences for model training, the samples were processed using the Z-score standardization method. This method is widely applied in deep learning, particularly in optimization algorithms based on gradient descent (Litjens et al., 2017). The basic idea is to perform a linear transformation on each feature value, resulting in a mean of 0 and a standard deviation of 1, ensuring that all features are on the same scale. The calculation formula is given as Equation (22):


$$Z=\frac{x-\mu }{\sigma }$$ (22)

where x is the original feature value, μ is the mean, σ is the standard deviation, and Z is the normalized value.

These standardization parameters (mean and standard deviation) were got by fitting *Z*-score scaler on this fold - training set, and then applied to transform this fold - training set, fold- validation set and independent test set.

In this study, *Z*-score standardization was applied not only to enhance training stability and reduce the risk of gradient explosion or vanishing, but also accelerate model convergence, improve the generalization ability and enhance the prediction performance for new data.

The SMOTE is one classical data augmentation strategy commonly applied to solve the problem of category distribution imbalance. This method can generate additional synthetic samples by interpolating between existing minority category samples, thereby increasing the amount of minority instances. In this study, SMOTE was exclusively applied to minority seizure samples within each fold - training set only. After finishing model training, validation and testing, these performance in 10-fold cross-validation was recorded and saved.

The detection of epileptic seizures and non-seizures is a typical binary classification problem. To alleviate model overfitting, an early stopping strategy was applied during training. We systematically compared model performance under different parameter combinations. Table 2 presents the averaged validation results of 10-fold cross-validation under different parameters. Using sensitivity as the primary evaluation indicator, while also considering accuracy and specificity, the optimal parameter combination was determined to be a learning rate of 0.001 and a batch size of 128. Under the optimal parameter settings, the model reached average accuracy, sensitivity, and specificity of 99.02, 98.80, and 98.75%, respectively, in 10-fold cross-validation.

**Table 2:** Model performance under different parameters (validation set).

Learning Rate	Batch Size	val_Accuracy (%)	val_Sensitivity (%)	val_Specificity (%)
0.01	128 256	94.33 97.21	92.67 96.13	94.89 97.90
**0.001**	**128** 256	**99.02** 99.00	**98.80** 98.06	**98.75** 98.12
0.0001	128 256	98.28 97.39	98.28 98.12	97.76 98.11

Bold values indicate the optimal parameter combination (learning rate = 0.001 and batch size = 128) and its corresponding best average validation results.

## 3 Results

### 3.1 UCI data seizure classification results

**Figure 4:**
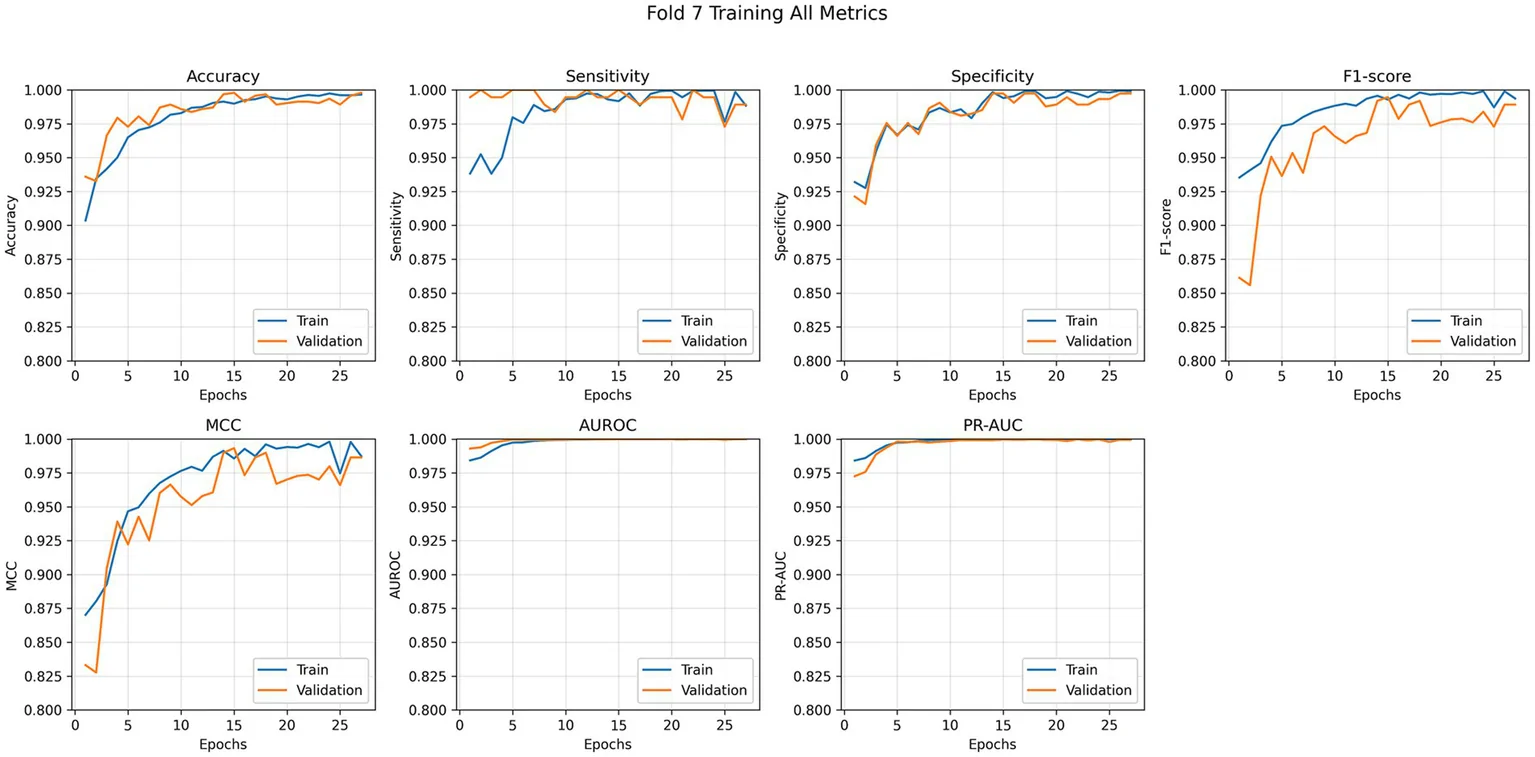
Epoch-wise training and validation curves of seven evaluation metrics (Accuracy, Sensitivity, Specificity, F1-score, MCC, AUROC, PR-AUC) from the optimal 7th stratified cross-validation fold. Blue lines represent training metrics, and orange lines represent validation metrics.

As shown in Table 3, The model with optimal parameters demonstrated robust performance in 10-fold cross-validation on the validation set. Sensitivity is used as the main evaluation metric for the performance of seizure detection model (Emami et al., 2019). As shown in Figure 4, the results of the best - performing model are showed in the 7th fold cross validation: the accuracy is 99.70%, the sensitivity is 99.54%, the specificity is 99.46%, the F1-score is 99.46%, the MMC is 99.32%, the AUROC is 99.99%, and the PR-AUC is 99.99%, for validation set.

**Table 3:** 10-fold performance with optimal parameters.

Fold number	Acc (%)	Sen (%)	Spe (%)	F1 (%)	MCC (%)	AUROC (%)	PR-AUC (%)
1	97.50	95.65	97.96	93.87	92.32	99.79	99.16
2	99.13	99.36	98.12	98.91	98.64	99.99	99.95
3	99.10	98.96	99.15	98.40	98.01	99.99	99.97
4	98.95	98.91	98.38	98.38	97.97	99.89	99.73
5	99.24	99.26	98.96	98.12	97.66	99.99	99.98
6	99.13	99.16	98.72	97.86	97.34	99.97	99.90
**7**	**99.70**	**99.54**	**99.46**	**99.46**	**99.32**	**99.99**	**99.99**
8	99.13	99.23	99.05	97.08	96.37	99.92	99.79
9	99.17	99.15	99.01	98.36	97.96	99.97	99.87
10	99.15	98.78	98.69	98.12	97.66	99.98	99.92
**Mean ± SD**	**99.02 ± 0.59**	**98.80 ± 1.13**	**98.75 ± 0.48**	**97.86 ± 1.53**	**97.33 ± 1.92**	**99.95 ± 0.07**	**99.83 ± 0.25**

Bold values indicate the optimal validation performance achieved in the 7th fold of the 10-fold cross-validation and the overall average performance (Mean ± SD) across all folds.

Finally, the test result got in 7th fold cross validation was thought to be this final classification testing result: a 99.23% accuracy, a 99.29% sensitivity, and a 99.34% specificity, and a 0.05% balance value (*Δ* Sens - Spec). These results confirm the effectiveness of the proposed hybrid network model based on CNN-Bi-LSTM-GRU and attention mechanism for epileptic seizure detection in EEG signals.

### 3.2 Ablation study

To investigate the contribution of each component in the proposed model and the data-balancing strategy (SMOTE), ablation experiments were conducted by progressively removing individual modules while maintaining the remaining network architecture unchanged (Paneru, 2026; Ru et al., 2024). Five simplified variants were constructed: (1) model without attention mechanism; (2) model without GRU; (3) model without Bi-LSTM; (4) model without CNN; and (5) model without SMOTE. The performance of these variants was average values across 10 cross-validation folds, evaluated and compared with the complete proposed model using Acc, Sen, Spe, F1-score, MCC, AUROC and PR-AUC.

There presents the averaged test results of 10-fold cross-validation in Table 4, the complete proposed model achieved the best overall performance across all evaluation metrics, with an accuracy of 99.13%, sensitivity of 98.86%, specificity of 99.20%, F1-score of 97.86%, MCC of 97.32%, AUROC of 99.95%, and PR-AUC of 99.83%. These results demonstrate that all these ablation variants produced inferior performance compared with the complete proposed model, confirming that each component (CNN, Bi-LSTM, GRU, the attention mechanism, and the SMOTE) contributes positively to the overall classification performance.

**Table 4:** Ablation study results of different model components and the SMOTE.

Model	Acc (%)	Sen (%)	Spe (%)	F1-score (%)	MCC (%)	AUROC (%)	PR-AUC (%)
Proposed	**99.13**	**98.86**	**99.20**	**97.86**	**97.32**	**99.95**	**99.83**
Without Attention	98.87	98.02	99.08	97.20	96.50	99.84	99.52
Without GRU	98.78	98.22	99.03	97.59	96.99	99.87	99.65
Without Bi-LSTM	99.01	98.59	99.10	97.52	96.90	99.85	99.73
Without CNN	98.94	98.22	99.12	97.38	96.74	99.84	99.61
Without SMOTE	99.00	98.24	99.20	97.54	96.93	99.86	99.65

Bold values indicate the performance of the complete proposed model, which achieved the best overall performance among all compared ablation variants.

## 4 Discussion

Compared to previous studies, traditional machine learning methods can achieve satisfactory accuracy on small datasets; however, their modeling ability remains limited when dealing with complex time series features and relies heavily on manual feature engineering. On the other hand, a single RNN network is easily influenced by the gradient vanishing problem when handling long-term dependency relationships, that resulting in a decline in detection accuracy. To further address the problems of insufficient local feature extraction and redundant noise interference in EEG signals, this study introduces 1D-CNN layers for adaptive extraction of subtle local waveform features and a temporal attention mechanism to enhance critical seizure-related features and suppress irrelevant noise. For these problems, this study employed a Bi-LSTM network to capture contextual temporal relationships in EEG signals, combined with a GRU to efficiently model temporal dynamics with limited parameters and computational cost. By integrating CNN, attention mechanism, and the two recurrent networks, we achieved a marked improvement in overall model performance. SMOTE was introduced to solve the category imbalance problem in the UCI dataset, and a 10-fold cross-validation strategy was applied to enhance the reliability of results.

As shown in Table 5, the proposed hybrid model demonstrates superior classification performance on the UCI dataset compared with most mainstream methods in recent years. Compared to single CNN, LSTM, or GRU models, as well as other conventional hybrid architectures without attention enhancement, the proposed method exhibits more superior performance across key evaluation indicators, including accuracy, sensitivity, and specificity. This proposed model reached not only a accuracy of 99.23%, but also a great sensitivity of 99.29%, reflecting a strong ability to identify seizure EEG and significantly reduce the risk of missed detection. Additionally, a specificity of 99.34% indicates that the model generates fewer false alarms during non-seizure periods. These key evaluation indicators are all important characteristics for clinical support and real-time monitoring, indicating our proposed method possessing good clinical practicability.

**Table 5:** Comparison of epileptic seizure detection methods on the UCI dataset.

Authors	Methods	No. of Layers	Accuracy (%)	SEnsitivity (%)	Specificity (%)	Balance (Δ Sens–Spec)
Kunekar et al. (2023)	Random Forest	—	97.70	92.80	99.00	6.2
Kode et al. (2024)	1D CNN	11	99.00	99.00	—	—
Kunekar et al. (2024)	LSTM	3	97.10	—	—	—
Britto et al. (2023)	Multi-dimensional CNN BiLSTM	13	99.61	97.42	99.35	1.93
Vinod and Dharmender (2023)	Modified Gated Recurrent Unit	5	98.84	97.10	-	-
Ahmad et al. (2026)	1D CNN-BiLSTM + TBPTT	11	99.41	98.99	98.80	0.19
Raibag et al. (2022)	Naive Bayes	-	96.00	94.00	96.00	2.00
Woodbright et al. (2021)	CNN	8	98.65	96.29	99.25	2.96
Rahman et al. (2021)	SVM	—	97.86	93.40	98.98	5.58
Shiva Shankar et al. (2021)	PCA with ANN	—	97.55	91.48	—	—
Kuang et al. (2024)	1D-CNN	7	98.70	97.53	98.98	1.45
Xu et al. (2020)	1D CNN-LSTM	13	99.39	—	—	—
**Us**	CNN-Bi-LSTM-GRU with attention mechanism	6	**99.23**	**99.29**	**99.34**	**0.05**

Bold values indicate the results obtained by the proposed method.

Besides, many models tend to favor most classes, often leading to “high specificity but low sensitivity,” that’s showed in Table 5. The imbalance between sensitivity and specificity, which can show bias towards positive or negative samples, were more easily overlooked. In contrast, our model’s nearly equal sensitivity and specificity (*Δ* = 0.05) suggests robust and almost unbiased classification, which is critical for clinical application with more clinical value.

These experimental results demonstrated that the proposed CNN-Bi-LSTM-GRU and attention-integrated hybrid model performed effectively in the epileptic seizure detection with EEG data.

Then, through the analysis of the ablation experiment results in Table 4, we observed when the attention mechanism was removed (Without Attention), the accuracy decreased from 99.13 to 98.87%, while the MCC decreased from 97.32 to 96.50%. In addition, the F1-score and PR-AUC also showed slight reductions. These results indicate that the attention mechanism effectively assigns adaptive weights to informative temporal features, enabling the model to emphasize discriminative EEG patterns associated with seizure events. After removing the GRU module (Without GRU), the model performance declined across several evaluation metrics, including sensitivity (from 98.86 to 98.22%) and MCC (from 97.32 to 96.99%). This suggests that the GRU module contributes to capturing sequential dependencies in EEG signals and complements the temporal representations learned by other network components. For the variant without the Bi-LSTM module (Without Bi-LSTM), the sensitivity decreased to 98.59%, while the MCC declined to 96.90%. The performance degradation demonstrates that the bidirectional temporal modeling capability of Bi-LSTM provides complementary information from both forward and backward directions, which is beneficial for identifying complex EEG seizure-related patterns. When the CNN module was removed (Without CNN), the accuracy decreased to 98.94%, and the MCC decreased to 96.74%. This decline indicates that the convolutional layers are effective in extracting local discriminative characteristics from raw EEG signals before high-level temporal modeling.

To further evaluate the contribution of the SMOTE-based data balancing strategy, an additional ablation experiment was conducted by removing SMOTE (Without SMOTE). Without SMOTE, the accuracy decreased from 99.13 to 99.00%, while the MCC decreased from 97.32 to 96.93%. Slight reductions were also observed in sensitivity, F1-score, AUROC, and PR-AUC. It should be noted that SMOTE was applied only to the training folds during cross-validation, while the validation and test data remained unchanged, ensuring a fair evaluation without introducing data leakage. These results indicate that SMOTE effectively alleviates the influence of class imbalance by generating synthetic minority samples during training, thereby improving the model’s generalization ability and enhancing overall classification performance.

Furthermore, MCC consistently exhibited more noticeable performance degradation than other evaluation metrics after removing individual components, indicating that the complete model provides more reliable classification performance, particularly under class-imbalanced conditions.

The combination of all these model components (CNN, Bi-LSTM, GRU, the attention mechanism) and the SMOTE enables the proposed framework to achieve superior and more robust EEG seizure detection performance.

## 5 Conclusion

In this study, a CNN-Bi-LSTM-GRU hybrid network model integrated with the attention mechanism was proposed to apply on the UCI epileptic seizure dataset. Combined with 1D-CNN-based local adaptive feature extraction and attention-based key feature enhancement, the experimental results demonstrated that this model can effectively capture the complex features in long time sequences while controlling computational cost. The SMOTE was applied to solve the data category imbalance problem. A 10-fold cross-validation was applied to ensure stable and reliable evaluation. The hybrid network model ultimately achieved a 99.23% accuracy, a 99.29% sensitivity, a 99.34% specificity, and the difference (sensitivity- specificity) of only 0.05%. These results confirmed the promising prospects of this hybrid model for EEG epileptic seizure detection.

Although our model achieves balanced performance on the UCI dataset, we acknowledge that this dataset consists of pre-processed single-channel EEG segments. Future validation on continuous multi-channel clinical recordings is needed to assess generalizability. Future work will further optimize and validate the model using multi-channel continuous clinical EEG recordings to explore its auxiliary application value in neurological disease monitoring.

## Data Availability

The original contributions presented in the study are included in the article/supplementary material, further inquiries can be directed to the corresponding author.
